# Prognostic marker CXCL5 in glioblastoma polyformis and its mechanism of immune invasion

**DOI:** 10.1186/s12885-023-11650-3

**Published:** 2024-01-29

**Authors:** Wangyang Yu, Minfeng Zhou, Huifang Niu, Jinxiao Li, Qiumeng Li, Xiaoyun Xu, Fengxia Liang, Chen Rui

**Affiliations:** 1 Union Hospital Affiliated to Tongji Medical College, Huazhong University of Science and Technology, Wuhan, China; 2 College of Food Science and Technology, Huazhong Agricultural University, Wuhan, China; 3 Suizhou Hospital of Traditional Chinese Medicine, Suizhou, China; 4 School of Acupuncture and Bone Injury, Hubei University of Chinese Medicine, Wuhan, China

**Keywords:** Glioblastoma multiforme (GBM), CXCL5, Immune invasion, Brain cancer, Immunoinfiltration

## Abstract

Glioblastoma multiforme (GBM) is the most aggressive brain cancer with a poor prognosis. Therefore, the correlative molecular markers and molecular mechanisms should be explored to assess the occurrence and treatment of glioma.WB and qPCR assays were used to detect the expression of CXCL5 in human GBM tissues. The relationship between CXCL5 expression and clinicopathological features was evaluated using logistic regression analysis, Wilcoxon symbolic rank test, and Kruskal–Wallis test. Univariate, multivariate Cox regression and Kaplan–Meier methods were used to assess CXCL5 and other prognostic factors of GBM. Gene set enrichment analysis (GSEA) was used to identify pathways associated with CXCL5. The correlation between CXCL5 and tumor immunoinfiltration was investigated using single sample gene set enrichment analysis (ssGSEA) of TCGA data. Cell experiments and mouse subcutaneous transplanted tumor models were used to evaluate the role of CXCL5 in GBM. WB, qPCR, immunofluorescence, and immunohistochemical assays showed that CXCL5 expression was increased in human GBM tissues. Furthermore, high CXCL5 expression was closely related to poor disease-specific survival and overall survival of GBM patients. The ssGSEA suggested that CXCL5 is closely related to the cell cycle and immune response through PPAR signaling pathway. GSEA also showed that CXCL5 expression was positively correlated with macrophage cell infiltration level and negatively correlated with cytotoxic cell infiltration level. CXCL5 may be associated with the prognosis and immunoinfiltration of GBM.

**Supplementary Information:**

The online version contains supplementary material available at 10.1186/s12885-023-11650-3.

## Introduction

Gliomas are primary brain tumors that develop from the glial stem or progenitor cells [1]. Gliomas are classified as WHO grades I-IV based on the 2016 World Health Organization (WHO) classification, of which grade IV is considered glioblastoma. Glioblastoma multiforme (GBM is the most aggressive primary intracranial tumor [2]. Although chemotherapy or radiotherapy combined with surgery is the major treatment for GBM, the 5-year survival rate of GBM patients is only about 5.6%, with an overall survival period of only 14.6 months [3]. Different gene mutations involved in GBM, such as IDH1/2, EGFR, ATRXp53, PI3K, PDGFRA, RAF, and IGF-1 are the key molecular markers in GBM. Nonetheless, targeted therapy or other treatments for the above molecules have not achieved satisfactory results [4]. Therefore, the signature molecules and underlying mechanisms for GBM should be explored to improve GBM treatment.

The immune microenvironment(TIM), as a significant component within the microenvironment of GBM, undoubtedly harbors signature molecules associated with GBM. TIM is closely related to the occurrence, invasion, and metastasis of tumors, and plays a vital role in the diagnosis, prevention, and prognosis of tumors [5]. Meanwhile, the GBM immune microenvironment is composed of glioma-associated immune cells, such as microglia, macrophages, and B cells, and immune regulatory factors that regulate the progression of glioma [6]. Furthermore, the tumor microenvironment (TME) is complex because multiple cell types, cytokines, and signaling pathways are involved in creating an immunosuppressive environment. Thus limiting immunotherapy against GBM (GBM-IT) [7, 8]. Also, immune checkpoint inhibitor (ICI)-based immunotherapy is associated with increased tumor mutation burden and the degree of immune cell infiltration within TME [9]. The establishment of tumor immunophenotypes involves a complex interplay between immune cell infiltration and genetic mutations.

Chemokines are central components of the TME, and previous research has identified several signature molecules closely associated with tumor initiation and progression [10]. Chemokines are small cytokines or signaling proteins secreted by cells inducing directed chemotaxis of nearby responding cells [11]. The chemokine CXC family binds to G protein-coupled receptors on target cells. CXCL5, the ligand of CXCR2, can mediate tumor cell migration and invasion and has many roles in colorectal cancer [12] and cervical cancer [13]. For example, CXCL5 can recruit immune cells to paracrine [12]. CXCL5 triggers tumor metastasis and promotes the formation of an immunosuppressive microenvironment [14]. CXCL5 also has a tumor-promoting effect and thus is crucial in tumor research and tumor immune microenvironment regulation, indicating that CXCL5 may be a key prognostic indicator for GBM. This study aims to elucidate the pivotal role of CXCL5 as a key molecular target in GBM diagnosis through a combination of bioinformatics analysis and experimental investigations. Additionally, it provides preliminary insights into the molecular mechanisms underlying GBM. The study serves as a foundational data source for subsequent research.

## Methods and materials

### Data acquisition and preprocessing

Gene expression RNA-Seq data of 112 GBM patients and 50 normal subjects, immune system infiltration, and relevant patient clinical information were obtained from The Cancer Genome Atlas (TCGA) database (https://portal.gdc.cancer.gov/). The RNAseq data were then converted from FPKM format to TPM format, retaining clinical data and RNAseq data. The data were further analyzed according to the publication guidelines provided by TCGA.

### Analysis of differentially expressed genes (DEG)

The expression data (HTseq-Counts) were divided into high and low expression groups based on median CXCL5 expressions, then further analyzed via unpaired Student's t-test in the DESeq2 R package (3.6.3). Adjusted *p* < 0.05 and |log2 fold change (FC)|> 1.5 were considered thresholds for DEG.

### Enrichment analysis

ClusterProfiler package in R (3.6.3) was used for Gene Ontology (GO) functional enrichment analysis and Gene Set Enrichment Analysis (GSEA). DEGs between high and low-expression groups were selected for further analysis. GO analysis includes cellular components (CC), molecular functions (MF), and biological processes (BP). GSEA is a computational method mainly used to determine whether a set of a priori-defined genes differ significantly and consistently in two biological states. In addition, the enriched pathways in each phenotype were classified based on normalized enrichment scores (NES) and adjusted *p*-values. C2. Cp.v7.2. symbols.gmt, and all. v7.2. symbols.gmt were used as the reference gene set for KEGG pathway C5 and. Gene sets with false discovery rate (FDR) < 0.25 and adjusted *p* < 0.05 were considered significantly enriched.

### Immune infiltration assay

The correlation between CXCL5 and 24 signature genes of immune cells was assessed using ssGSEA via the GSVA package in R. Immune infiltration was then systematically analyzed. Spearman correlation and Wilcoxon rank sum test were used to analyze the infiltration of immune cells between the high and low-expression groups.

### Protein–protein interaction network (PPI)

The interacting Genes Database retrieval tool (http://string-db.org) and Cytoscape software (version 3.8.1) were used to assess protein–protein interaction (PPI) network of co-regulated DEGs and the functional interactions between proteins. The composite score threshold for interactions was 0.7. The database has a composite score for each pair of protein relationships distributed between 0 and 1. The higher the total score, the more reliable the PPI relationship.

### Validation analysis

Differential CXCL5 expression between HCC and non-tumor tissues in three RNAseq datasets obtained from the GEO database was analyzed. The Kaplan–Meier (KM) plotter can be used to assess the impact of genes on survival in cancer types. Sources of databases include GEO, EGA, and TCGA. The KM plotter is mainly used to discover and validate survival biomarkers based on meta-analysis. Herein, the relationship between CXCL5 expression and GBM patient survival days was analyzed using KM plotter, then visualized in the KM survival plot. A log-rank *p*-value < 0.05 was considered statistically significant.

### Construction of mouse subcutaneous tumor model

The mice were adapted to the environment for 1–2 weeks, depilated, marked clearly with a permanent marker, and anesthetized before tumor cell injection. The glioma cells GL261 and their stable transfection cell lines were prepared according to the number of injected mice. Each cell line was prepared for 1.5 h. Cells in exponential growth phase were detached from tissue culture flasks or dishes using trypsin or other suitable enzyme preparation (Accutase) and kept in 100% cell culture medium on ice to maintain their viability. The tumor cells were subcutaneously injected based on the number of mice) allow 2–3 min for subcutaneous injection; 4–5 min if anesthesia is required). The cells were transferred near the animals while on ice to keep them alive. Tumor growth was measured daily after tumor cell injection. All animal experiments followed the ethical principles of experimental animals of Huazhong University of Science and Technology, and received ethical approval.

### Cell cultures

GBM cell lines transfected with GL261 were sourced from the U.S. Typical Culture Preservation Center (ATCC, Manassas, Virginia, USA). The cell lines were put in Dulbecco-modified Eagle medium supplemented with 10% fetal bovine serum, then placed in an incubator (37 °C, 5% CO2). The cell lines were verified using short tandem repeat analysis via the GenePrint 10 system at Genome Australia. Lookout Mycoplasma PCR detection kit was used to confirm that the cell lines were free of mycoplasma.

### Construction of lentiviral stably transfected cell lines

The optimal lentivirus multiplicity of infection (MOI) (the ratio of the number of viruses to cells at infection time) was determined. The drug screening concentration of the target cell was also determined. Different cells have different screening concentrations for the same antibiotics, while the same cells have different optimal concentrations for different antibiotics. The target cells were infected with lentivirus. The antibiotic screening was started 24 h after infection, and the medium was changed after the death of antibiotic-positive cells.The mRNA levels and protein levels were detected using qRT-PCR method and Western blot or cell immunofluorescence technology, respectively.

### Western blot

Total proteins were extracted from tissues and cell lines via western blot (WB) analysis using tissue extraction reagents and cell extraction buffers (Beyotime). Total proteins were also purified and qualitatively evaluated via (WB) analysis. The total protein (30 µg) was transferred to 12% graded sodium dodecyl sulfate–polyacrylamide gel electrophoresis gel to separate proteins with different molecular weights, then transferred to nitrate cellulose membrane for antigen–antibody reaction. The membrane was then sealed with TBST containing 5% skimmed milk for 1 h, then incubated with CXCL5(1:1,000; ABclonal), CD68(1:1,500; ABclonal), and β-actin (1:1,000; ABclonal). The membrane was also incubated with goat anti-rabbit secondary antibody at 37°C for 2 h.

### RNA extraction and quantitative real-time polymerase chain reaction (qPCR)

Total RNA was extracted from cancer tissue using Trizol (Takara) and matched to normal tissue and cell lines.The cDNA was then synthesized via PrimeScript™ RT kit following the manufacturer's instructions. The following specific primers were used: GAPDH (mouse), SIRT1 (mouse), FOXQ1 (mouse), ATG16L (mouse), Becline1 (mouse), p62 (mouse), and LC3 (mouse). QuantiTest SYBR-Green Polymerase Chain reaction (PCR) reagent boxes (Norvizan Biological Technology Co., Ltd., Nanjing, China) were used for real-time PCR via Applied Biosystems 7500 (Applied Biosystems, Foster City, CA, USA). GAPDH was used as a standardized control for all the mRNAs above. The primers used for qRT-PCR are shown in Table 1.

**Table 1:** Demographic and clinicopathological parameters of high and low CXCL5 expression group patients with GBM

Characteristic	Low expression of CXCL5	High expression of CXCL5	*P*
N	84	84	
T stage, n (%)			0.091
T1	46	44	
T2	23	25	
T3	10	9	
T4	5	6	
N stage, n (%)			0.668
N0	79	80	
N1	5	4	
M stage, n (%)			0.774
M0	81	79	
M1	3	5	
Pathologic stage, n (%)			0.189
Stage I	48	48	
Stage II	21	23	
Stage III	9	7	
Stage IV	6	6	
Gender, n (%)			0.598
Female	42	40	
Male	42	44	
OS event, n (%)			0.014
Alive	52	40	
Dead	32	44	
Vascular invasion, n (%)			0.077
No	61	58	
Yes	23	26	
Histologic grade, n (%)			0.801
G1	23	20	
G2	48	49	
G3	11	12	
G4	2	3	
Age, median (IQR)	65.8 (51, 66)	66.1 (50, 73)	0.401
AFP(ng/ml), median (IQR)	8.3 (4, 108.5)	25.86 (9,804.75)	< 0.01
BMI, median (IQR)	26.24 (22.37, 28.47)	22.56 (19.36, 31.01)	0.029

### Immunofluorescence and immunohistochemistry

We utilized immunofluorescence and immunohistochemistry techniques to investigate specific protein expression patterns in our experimental samples. For immunofluorescence, we initially prepared our samples by fixing tissue sections or cell cultures with appropriate fixatives, permeabilizing them using a permeabilization buffer, and blocking nonspecific binding with a blocking solution. Following this, we incubated the sections or cells with specific primary antibodies overnight, carefully selected for their target protein specificity. Subsequently, we washed the samples to remove unbound antibodies, followed by incubation with secondary antibodies conjugated to fluorophores, matching the host species of the primary antibodies when necessary. Optional nuclear staining was conducted using a nuclear counterstain such as DAPI or Hoechst. To preserve the samples and prevent photobleaching, coverslips were applied using a suitable mounting medium. Finally, we captured immunofluorescent signals through a fluorescence microscope or confocal microscope for image acquisition. The antibodies used in the study were CD68 (abcam, 259875) and Microtubulin (abcam, 357895).

In the case of immunohistochemistry, we began by sectioning paraffin-embedded tissue blocks into thin slices and mounting these sections onto glass slides. Deparaffinization was achieved either through oven heating or the use of xylene, followed by rehydration via graded ethanol solutions. Antigen retrieval was performed by subjecting the sections to heat in an appropriate buffer solution to expose antigenic sites. To prevent nonspecific antibody binding, sections were blocked with a suitable blocking solution. Specific primary antibodies were then applied and incubated for an appropriate duration, allowing them to bind to their target antigens. Unbound primary antibodies were removed through washing with a buffer solution, followed by incubation with secondary antibodies, typically conjugated to an enzyme or chromogen, for signal amplification. Detection was achieved by applying a chromogenic substrate to visualize antibody-antigen binding, resulting in a visible signal. Optional counterstaining with hematoxylin or eosin was performed to visualize tissue structures. Finally, coverslips were applied using mounting media to preserve the stained sections, and stained sections were examined and imaged under a light microscope. These methods enabled the assessment of specific protein expression and localization, providing valuable insights into the investigated biological processes. The antibodies used in the study were CD68 (abcam, 259875) and Microtubulin (abcam, 357895).

### Statistical analysis

R 3.7.1 was used to process data obtained from TCGA. Comparing CXCL expression levels between GBM and normal groups was conducted using the Wilcoxon rank sum test and the Wilcoxon sign rank test. Welch one-way ANOVA, followed by Bonferroni correction or t-test was used to assess the correlation between CXCL expression and clinicopathological factor grade. Univariate logistic regression, Fisher exact test, and normal /adjusted Pearson κ2 were used to evaluate the effect of clinicpathological factors on CXCL expression. Univariate and multivariate Cox regression analyses were used to evaluate the prognostic value of CXCL expression and other clinicopathological factors on overall survival (OS). All variables in the univariate analysis were included in the multivariate analysis. The prognostic value of CXCL was evaluated using KM curve. The Hazard Ratio(HR) of OS and disease-specific survival (DSS) in individuals was analyzed using univariate Cox proportional hazard regression. HR for each factor was estimated by measuring HR with a 95% confidence interval (CI).

## Results

### Elevated CXCL5 Expression in GBM

**Fig. 1:**
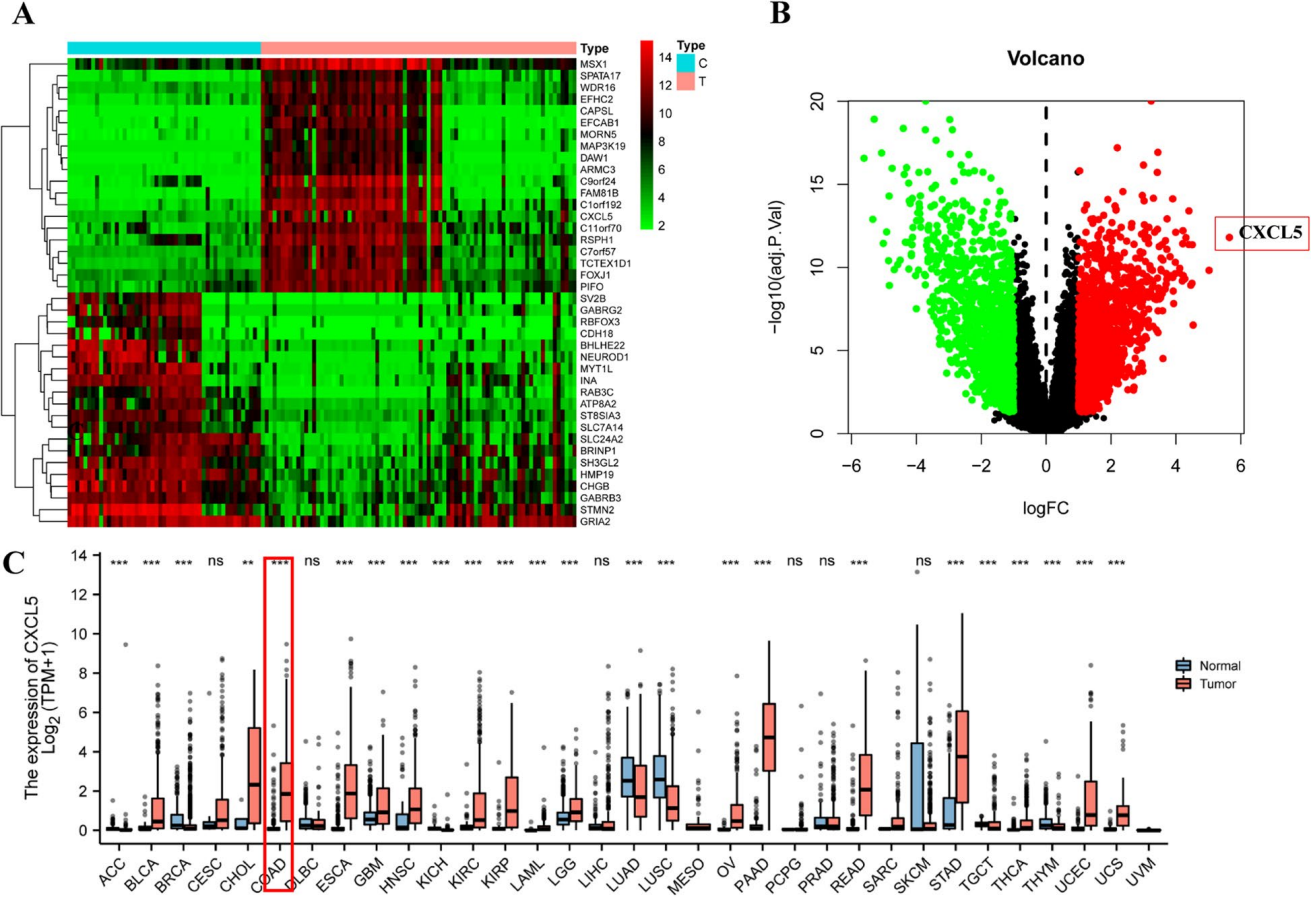
CXCL5 expression in Glioblastoma multiform (GBM). **A** Heat map and (**B**) scatterplot showing differential gene expression analysis in tumor tissues and adjacent normal tissues. CXCL5 was highly expressed in GBM tissues. **C** Pan-cancer analysis of CXCL5 expression in various malignant tumors. C: control; T: tumor; Asterisks ** and *** represent *p* < 0.01 and *p* < 0.001, respectively

Malignant tumors are diseases caused by the accumulation of gene mutations [1]. In this study, gene expression datasets of GBM and normal brain tissues were retrieved from the GEO database for differential analysis. The GSE50161 dataset was utilized for differential gene expression analysis, revealing an upregulation of CXCL5 in GBM tissues (Fig. 1A and Fig. 1B). Additionally, we compared the differential expression of CXCL5 molecules in various tumor tissues and normal tissues using the TCGA database (Fig. 1C). Consistently, CXCL5 was found to be upregulated in various tumor types, including Adrenocortical carcinoma (ACC), Cholangiocarcinoma (CHOL), Colon adenocarcinoma (COAD), and GBM, underscoring the upregulation of CXCL5 in GBM tissues.These findings suggest a potential association between the abnormal expression of CXCL5 and the development and progression of GBM. These data provide crucial clues for further investigating the functional role of CXCL5 in GBM and its potential therapeutic implications.

### Patients exhibiting elevated CXCL5 expression experience an unfavorable prognosis

GBM patients (112) were divided into low-expression (*n* = 56) and high-expression groups (*n* = 56) based on CXCL5 expression. The clinical data of the above patients, including gender, race, age, karnofsky performance score, IDH status, OS event, DSS event, and PFI events were also collected (Table 1). Fisher's test showed that CXCL5 was significantly associated with IDH status (*p* = 0.038), OS event (*p* = 0.037), DSS event (*p* = 0.041), and PFI event (*p* < 0.001). However, CXCL5 expression was not significantly associated with other clinicopathological features. The researchers aim to further elucidate the role of CXCL5 in GBM at the genetic level and explore it comprehensively through a combination of bioinformatics analysis and experimental investigations.

### Identification of Differentially Expressed Genes (DEGs) Related to CXCL5 in GBM

**Fig. 2:**
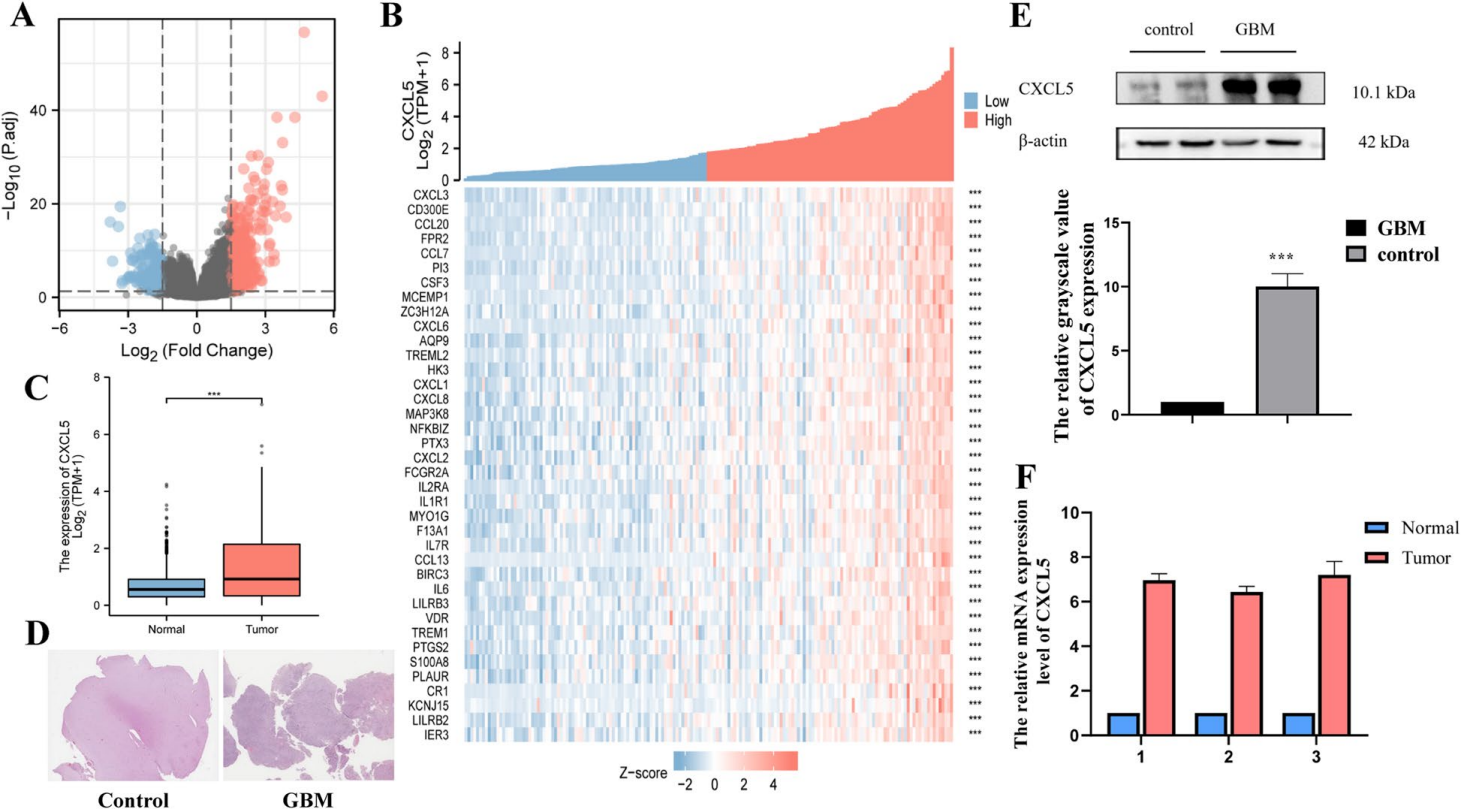
Analysis of differentially expressed genes (DEG) in GBM. **A** The volcano plot showing differentially expressed genes. **B** Histogram of differential expression of CXCL5 between GBM group and normal group. **C** Heat maps of 38 genes highly associated with CXCL5. **D** Immunohistochemical showing CXCL5 expression in human GBM tissues. **E** Western Blot showing CXCL5 expression in human GBM tissues. **F** RT-qPCR showing CXCL5 expression in human GBM tissues. C: control; T: tumor; Asterisks **p < 0.01and *** represent *p* < 0.01 and *p* < 0.001, respectively

DEGs were identified via single-gene differential analysis of CXCL5 on the GBM dataset in the TCGA database at |logFC|< 1.5 and adjusted *p* < 0.001. A total of 408 DEGs were identified (263 up-regulated and 145 down-regulated) via HTSEQ-Counts data of CXCL5-related genes. The visualization ofßå© is shown in Fig. 2A, where blue and red in the volcano plot represent up-regulated and down-regulated genes, respectively. Single-gene correlation analysis was used to detect the genes with the highest correlation with CXCL5 in the TCGA GBM dataset (*n* = 38, correlation coefficient > 6.5) (Fig. 2B). TCGA database dataset was combined with human GBM postoperative tissues to clarify the expression of CXCL5 in GBM tissues and normal tissues. Unpaired differential expression analysis between the normal group and GBM group showed that CXCL5 expression was significantly higher in tumor tissues than in normal tissues (*p* < 0.05) (Fig. 2C). Further analysis was conducted using the tumor tissue and paracancerous tissue of GBM patients. Immunohistochemical results confirmed that CXCL5 expression was significantly higher in GBM tissues than in adjacent tissues at the organizational level (*n* = 3, *p* < 0.01) (Fig. 2D). Furthermore, WB results showed that CXCL5 expression was higher in human GBM tissues than in normal tissues at the protein level (*n* = 3, *p* < 0.001) (Fig. 2E). RT-qPCR results also showed that CXCL5 was up-regulated in human GBM tissues (*n* = 3, *p* < 0.001) (Fig. 2F). Meanwhile, CD300E, CCL20, FPR2, and other molecules were highly correlated with the high CXCL5 expression in GBM tissues, providing preliminary prediction of targets for further mechanism research.

### Pathway enrichment analysis highlights CXCL5's Role in GBM

**Fig. 3:**
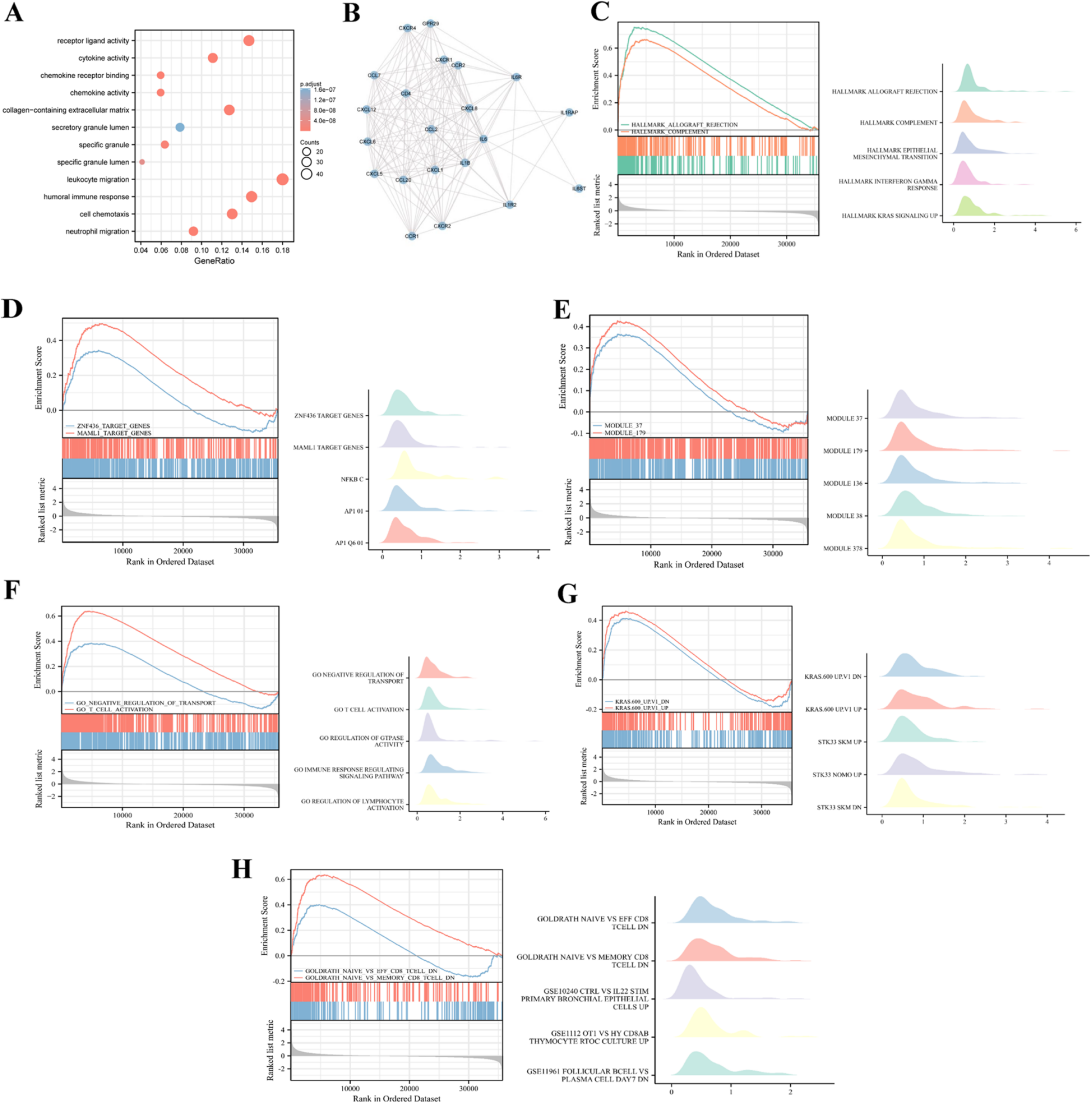
Enrichment analysis of CXCL5 in GBM. **A** Biological process enrichment analysis of CXCL5-related genes. **B** A network of CXCL5 and its 28 potential co-interaction proteins. **C**–**H** Enrichment analysis result from GSEA in MsiDB dataset. The thresholds for significant enrichment are false discovery rate (FDR) < 0.25 and p.adjust < 0.05

GO analysis indicated that CXCL5-related DEGs were associated with receptor ligand activity, cytokine activity, chemokine receptor binding, chemokine activity, collagen-containing extracellular matrix, specific granule, specific granule lumen, leukocyte migration, humoral immune response, cell chemotaxis and neutrophil Migration (Fig. 3A). Meanwhile, the STRING database (https://string-db.org/) was used to analyze the correlation network and the degree of co-expression relationship within the CXCL5-related DEGs. CXCL5 was significantly correlated with CD4, IL6, IL1B, CCR1, and CXCL subunits (Fig. 3B). The biological function of CXCL5 was further evaluated via GSEA analysis in MsiDB dataset (https://www.gsea-msigdb.org/). The differences between the low and high-expression groups were assessed to identify hallmark gene sets, regulatory target gene sets, computational gene sets, ontology gene sets, oncogenic signature gene sets, and immunologic signature gene sets. Analysis of hallmark gene sets, a hypergene set consisting of multiple known gene sets that correspond to multiple bases of other classes of genes, revealed that hallmark allograft rejection, hallmark complement, hallmark epithelial-mesenchymal transition, hallmark interferon-gamma response and hallmark KRAS signaling were correlated with CXCL5 (Fig. 3C). Analysis of regulatory target gene sets, potential targets for transcription factors or micro-RNA regulation, revealed that ZNF436 target genes, MAML1 target genes, NFKB CAP1 01 and AP1 Q6 were the potential targets of CXCL5 (Fig. 3D). Analysis of computational gene sets revealed that module37, module179, module136, module38, and module378 were significantly correlated with CXCL5 (Fig. 3E). Computational gene sets were defined by large cancer-oriented microarray data. Analysis of ontology gene sets, a set of genes annotated by the same ontology term, revealed that CXCL5 was correlated with negative regulation of transport, T cell activation, regulation of GTPase activity, immune response regulating signaling pathway, and regulation of lymphocyte activation (Fig. 3F). Analysis of oncogenic signature gene sets, a set of genes characterizing cellular pathways commonly misregulated in cancer, revealed that dysregulated cellular pathways in GBM included KRAS.600 UP.V1 DN, KRAS.600 UP.V1 UP, STK33 SKM UP, STK33 NOMO UP and STK33 SKM DN (Fig. 3G). Analysis of immunologic signature gene sets, a set of genes representing cellular states and disturbances within the immune system, revealed that CXCL5 is critical in immune regulation of EFF CD8 T cell, memory CD8 T cell, and IL22 and stimulates primary bronchial epithelial cells, Hy CD8AB thymocytes and plasmocyte (Fig. 3H). These results indicate that CXCL5 expression is significantly associated with the pathways regulating T-cell activation, KRAS-related molecules, and IL22-related molecules.

### The correlation between CXCL5 expression and immune infiltration

**Fig. 4:**
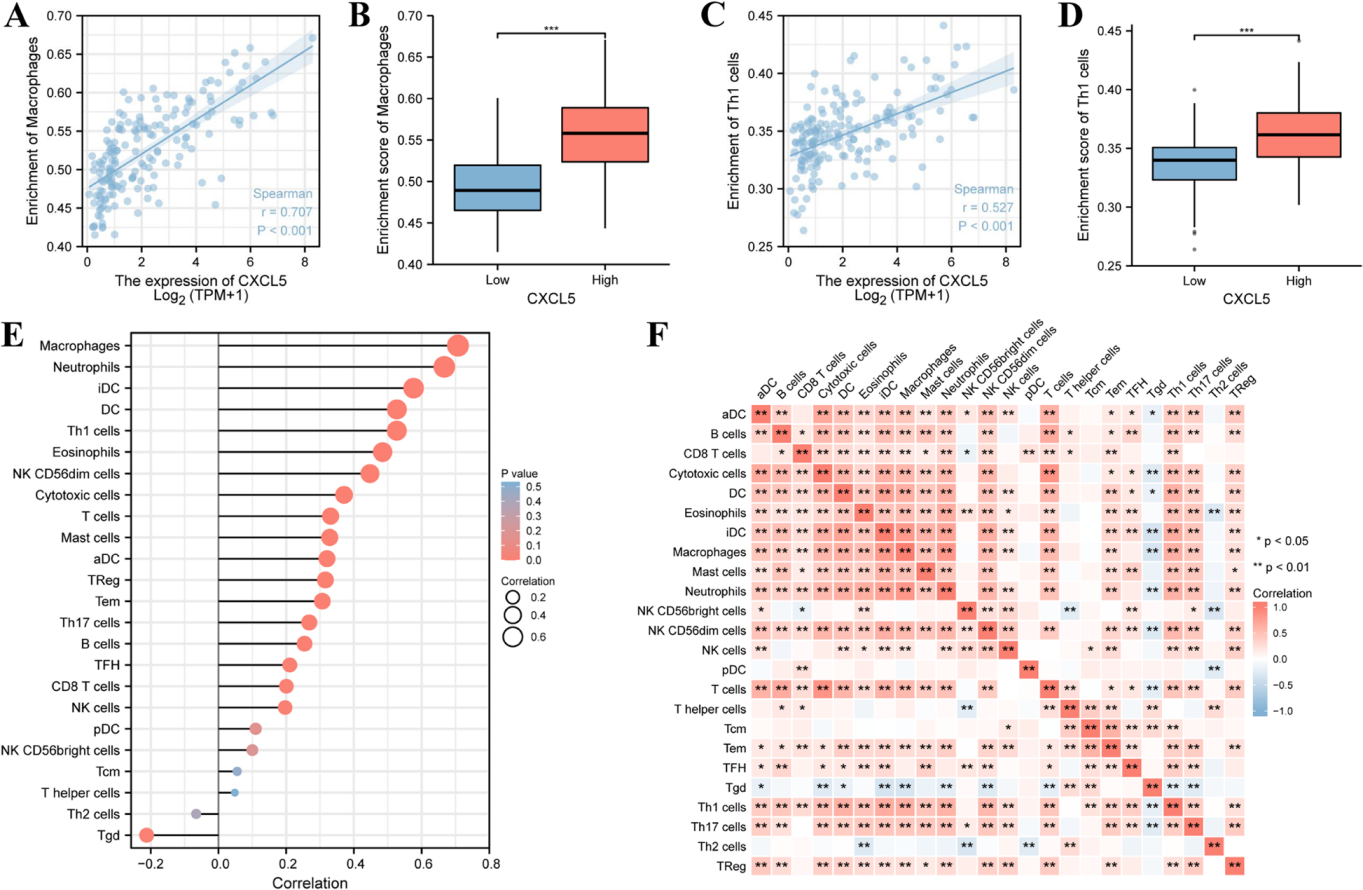
Relationship between differential expression of CXCL5 and immune infiltration. **A** The relationship between CXCL5 expression and macrophage expression. **B** The relationship between high and low expression groups with macrophage infiltration level. **C** The relationship between CXCL5 expression and Th1 cell expression. **D** The relationship between high and low expression group with Th1 cell infiltration. **E** The relationship between CXCL5 expression and the relative abundance of 24 immune cells. **F** Heat map of 24 immuno-infiltrated cells in GBM. Asterisks *, **, and *** represent *p* < 0.05, *p* < 0.01, and *p* < 0.001, respectively

Results indicated that CXCL5 is closely related to the immune system in GBM. Therefore, CXCL5 may play a key role in GBM through immune infiltration based on the characteristics of immune infiltration in GBM [2]. Herein, the correlation between GBM-associated CXCL5 expression in the TCGA database and immune cell infiltration levels was quantified as ssGSEA scores using Spearman correlation analysis. Infiltration analysis of all immune cells revealed that various CXCL5-associated immune cells were involved in the immune infiltration process of GBM (Supplementary Figure S1). Furthermore, CXCL5 expression was positively correlated with macrophage cell infiltration level (Spearman R = 0.707, *P* < 0.001) (Fig. 4A), and this correlation significantly increased in the high expression (*P* < 0.001) (Fig. 4B). In addition, Th1 cells showed the same trend (Spearman R = 0.527, *P* < 0.001) (Fig. 4C, D). In summary, macrophages, neutrophils, iDC, DC, Th1 cells, eosinophils, NK CD56dim cells, NK CD56dim cells, T cells, mast cells, aDC, TReg, Tem, Th17 cells, B cells, TFH, CD8 T cells, and NK cells were positively associated with CXCL5-related immune infiltration in GBM. In contrast, Th2 cells and Tgd cells were negatively correlated with CXCL5-related immune infiltration in GBM (Fig. 4E). These results suggest that CXCL5 plays a key role in the immune infiltration of GBM. The degree of correlation of the proportions of 24 different tumor-infiltrating immune cell subsets was expressed using heatmaps (Fig. 4F).

### The correlation between CXCL5 expression and clinical prognosis of GBM

**Fig. 5:**
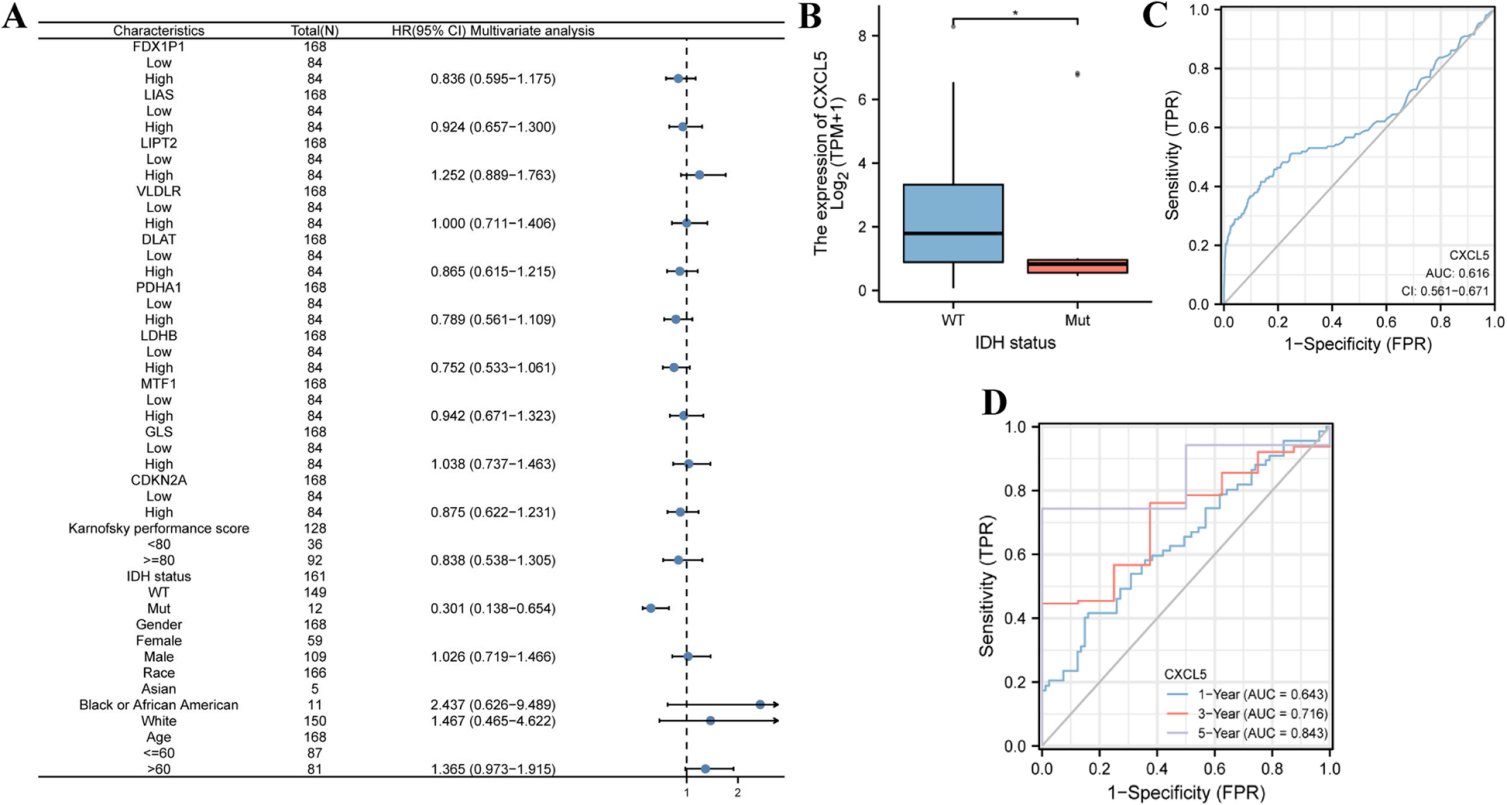
The prognostic value of CXCL5 in GBM. **A** Multivariate Cox regression visualized in the forest plot. **B** IDH stage. **C** Diagnostic ROC curve of CXCL5. **D** Time-dependent ROC curve of CXCL5

Univariate Cox regression suggested that IDH status (*p* = 0.002), age (*p* = 0.042), and CXCL5 (*p* < 0.01) were associated with poor prognosis in GBM (Table 2). Multivariate Cox regression revealed that IDH stage (*p* = 0.003) and CXCL5 (*p* < 0.01) were independent prognostic factors for OS (Fig. 5A). Logistic regression analysis showed that CXCL5 was significantly correlated with IDH stage (*P* < 0.05) (Fig. 5B), while gender, race, age, and karnofsky performance score were not correlated with IDH stage (Supplement Fig. 2 and Table 3). ROC analysis supported the diagnostic accuracy of this score (AUC = 0.616, 95% CI: 0.561–0.671) (Fig. 5C). Time-dependent ROC analysis was used to evaluate the time-dependent accuracy of CXCL5 in predicting OS at 1, 3, and 5 years (Fig. 5D).

**Table 2:** CXCL5 expression correlated with clinicopathological characteristics analyzed by logistic regression

Characteristics	Total (N)	Odds ratio (OR)	*p*-Value
T stage (T2 and T3 and T4 vs. T1)	168	1.305(0.985–2.354)	0.021
N stage (N1 vs. N0)	172	3.447(1.568–3.889)	0.386
N stage (N1 vs. N0)	132	0.886(0.458–1.568)	0.944
Pathologic stage (Stage III and Stage IV vs. Stage I and Stage II)	128	1.251(0.986–2.033)	0.158
Histologic grade (G3 and G4 vs. G1 and G2)	176	1.748(1.048–2.355)	0.018
Vascular invasion (Yes vs. No)	158	1.528(1.088–2.417)	0.048
AFP (ng/ml) (> 400 vs. ≤ 400)	168	1.058(0.735–1.686)	0.168
Albumin (g/dl) (≥ 3.5 vs. < 3.5)	169	0.844(0.408–1.590)	0.294
Tumor status (with tumor vs. tumor-free)	172	1.568(1.120–2.322)	0.318

**Table 3:** Univariate and multivariate analyses of clinical pathological parameters in CRC patients

Characteristics	Total (N)	Univariate analysis		Multivariate analysis	
		Hazard ratio (95% CI)	*p*-Value	Hazard ratio (95% CI)	*p*-Value
Age	284				
≤ 60	102	Reference			
> 60	182	1.058(0.838–1.799)	0.291	1.420 (0.845–2.565)	0.334
Gender	284				
Female	134	Reference			
Male	150	0.755 (0.458–1.334)	0.218	0.8585(0.418–1.708)	0.899
Histologic grade	286				
G1	41	Reference			
G2	68	1.224 (0.586–1.996)	0.598	0.825 (0.437–1.822)	0.508
G3-G4	187	1.222 (0.710–2.103)	0.484	1.041 (0.685–1.996)	0.994
T stage	248				
T1 and T2	158	Reference			
T3 and T4	90	2.052 (1.661–3.241)	0.014	2.255 (1.041–4.014)	0.011
M stage	268				
M0	264	Reference			
M1	4	1.589 (1.007–3.288)	0.012	1.085 (0.865–2.583)	0.331
N stage	258				
N0	253	Reference			
N1	3	1.156 (0.855–2.609)	0.344	1.186 (0.685–2.968)	0.481
CXCL5	336	1.058 (0.755–1.969)	0.004	1.035 (0.758–1.958)	0.028
Pathologic stage	308				
Stage I	148	Reference			
Stage II- IV	160	1.256 (1.008–2.668)	0.002	1.355 (0.868–2.055)	0.314

**Fig. 6:**
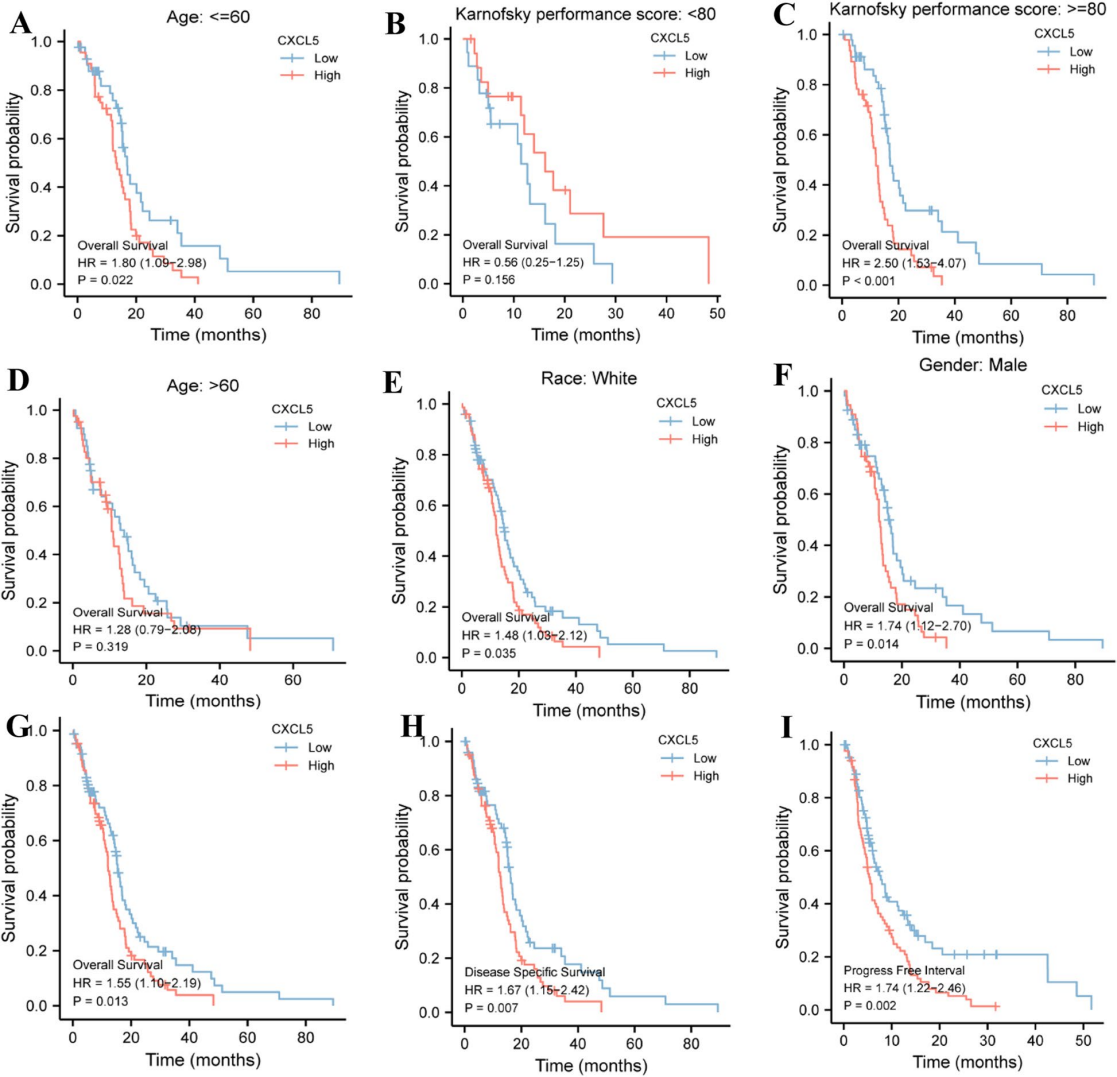
The prognostic value of CXCL5 in different subgroups. **A-F** of the relationship between CXCL5 and OS in different subgroups. **G-I** The prognostic value of CXCL5 in OS, DSS, and PFI of GBM

The K-M survival curve was drawn using survminer software package in R to evaluate the prognostic value of CXCL5 in GBM patients. GBM patients were divided into high and low expression groups based on the median CXCL5 expression. The high expression group was significantly correlated with the poor prognosis. Age le < 60 years (HR = 1.80 (1.09 − 2.98), *P* = 0.022) (Fig. 6A-B), Karnofsky performance score ≥ 80 (HR = 2.50 (1.53 − 4.07)), *P* < 0.001) (Fig. 6C-D), white race (HR = 1.48 (1.03 − 2.12), *P* = 0.035) (Fig. 6E), and male gender (HR = 1.74 (1.12 − 2.70), *P* = 0.014) (Fig. 6F) were significantly correlated with the poor prognosis. Also, the high expression group was strongly correlated with poor OS (HR = 1.55 (1.10 − 2.19), *P* = 0.013), DSS (HR = 1.67 (1.15 − 2.42), *P* = 0.003), and PFI (HR = 1.74 (1.22 − 2.46), *P* = 0.002) (Fig. 6G-I) (Table 4).

**Table 4:** The primer sequence information of qPCR

Gene name	Forward	Reverse
CXCL5	5'-ATCGGCTACGTAGCTGATCG-3'	5'-CGATCGACTAGCTTACGATG-3'
CD68	5'-GCCATGACGTACGTGACTGC-3'	5'-GCATGCTAGCATGCTAGCAT-3'
MARCO	5'-TACGTGCTAGCATGCATGCT-3'	5'-AGCTACGTAGCTAGCTAGCT-3'
IFN-γR1	5'-ATCGATCGATCGATCGATCG-3'	5'-GATCGATCGATCGATCGATC-3'

### CXCL5 promotes GBM cell progression by inhibiting macrophage and Th1 immune infiltration

**Fig. 7:**
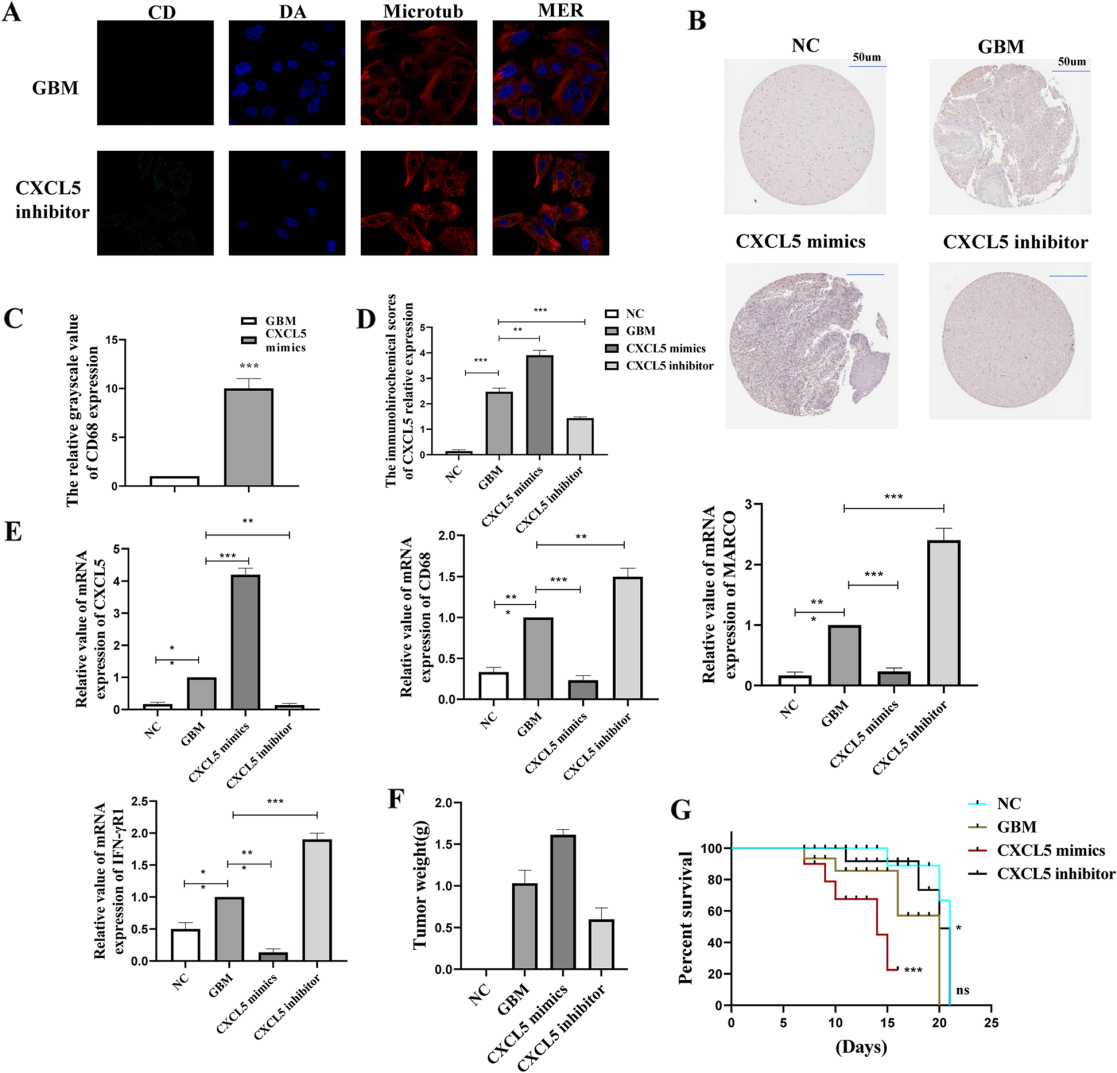
CXCL5 promotes GBM cell progression by inhibiting macrophage and Th1 immune infiltration. **A** Immunofluorescence showing expression of macrophage marker CD68 in mouse brain tissue after CXCL5 inhibition (statistical values are shown in **C**); **B** Mouse normal brain tissue under various treatments. The expression of CXCL5 in transplanted tumors (statistical values are shown in **D**); **E** RT-qPCR showing the expression of CXCL5, CD68, MARCO, and IFN-γR1;Subcutaneous tumor was injected into cells of each group to construct a mouse subcutaneous tumor model, and the subcutaneous tumor weight of each group was weighed (**F**) and the results of the mouse survival cycle of each group were analyzed after the third week (**G**). Asterisks *, **, and *** represent *p* < 0.05, *p* < 0.01, and *p* < 0.001, respectively

Further analysis showed that macrophages and Th1 were crucial in GBM immune infiltration, and were negatively correlated with CXCL5 expression. CXCL5-overexpressed and suppressed GL261 cell lines were constructed through stable lentiviral transfection to verify the results. Mouse subcutaneous tumor models were also constructed through injection of cells with different CXCL5 expressions. Immunofluorescence detection confirmed that the infiltration of macrophages increased in the tumor tissues of mice after CXCL5 knockout (Fig. 7A and C). immunohistochemical analysis showed that the CXCL5 expression in normal mouse brain tissue and mouse xenograft tumors was consistent with the hypothesis (Fig. 7B and D).The qPCR tests were performed to further verify the above results at the genetic level, and similar conclusions were achieved (Fig. 7E). Many studies have shown that CXCL5 promotes GBM cell progression by inhibiting macrophage and Th1 immune infiltration. Subcutaneous tumor was injected into cells of each group to construct a mouse subcutaneous tumor model, and the subcutaneous tumor weight of each group was weighed(Fig. 7F) and the results of the mouse survival cycle of each group were analyzed after the third week(Fig. 7G). In conclusion,CXCL5 promotes GBM cell progression by inhibiting macrophage and Th1 immune infiltration.

## Discussion

Glioblastoma multiforme (GBM) stands as one of the most common and aggressive forms of brain cancer [15]. Its clinical manifestations typically start with mild symptoms, gradually progressing to more severe conditions, with some signs indicating acute illness [16]. The conventional treatment approach for GBM patients involves surgical resection, followed by a combination of radiation therapy and chemotherapy. Unfortunately, these traditional therapies have not yielded significant improvements in patient survival rates, leaving the overall mortality rate disturbingly high [17]. In light of these challenges, immunotherapy has emerged as a promising avenue for cancer treatment, exhibiting effectiveness against various aggressive cancer types [18]. Given the formidable nature of GBM, it becomes imperative to delve into the molecular markers and underlying mechanisms driving its onset, while concurrently identifying potential therapeutic targets. A substantial body of research has indicated that the presence and functionality of CD8 + tumor-reactive proliferating T cells, particularly their capacity to modulate IFN-γ immunoregulation within the tumor microenvironment, can markedly impact patient prognosis [10]. Nevertheless, the intricate mechanisms underlying these observations remain partially elucidated and warrant further investigation.

In this pursuit, bioinformatics methodologies have gained significant traction, proving to be cost-effective and efficient tools for identifying tumor-related molecules and unraveling tumor immunity. They have played a pivotal role in laying the theoretical groundwork for diverse cancer types [19, 20]. Leveraging the extensive resources of The Cancer Genome Atlas (TCGA) database, we accessed gene-expression RNA-Seq data from 112 GBM patients and 50 normal controls, accompanied by immune cell infiltration profiles and clinical insights. Our analysis pinpointed CXCL5 as a differentially expressed gene, prompting us to conduct comprehensive enrichment analyses, immune infiltration assessments, protein–protein interaction network (PPI) investigations, and validation studies, all aimed at deciphering the mechanisms intertwined with CXCL5. The results unequivocally identified CXCL5 as the signature gene associated with GBM. Strikingly, high CXCL5 expression in GBM exhibited a negative correlation with patient prognosis. Furthermore, our findings suggested that CXCL5 could potentially modulate GBM progression by influencing immune infiltration patterns.

However, it is vital to acknowledge several limitations within our study. Firstly, due to experimental constraints, we were unable to delve deeper into the mechanisms underpinning our observations [21–24]. Consequently, the role of CXCL5 in GBM remains only partially understood, and the associated target molecules await further elucidation. Additionally, given the complexity of tumor mechanisms, our choice of models and initial exploration of mechanisms may not have fully addressed the multifaceted nature of GBM [25–27]. Therefore, future research endeavors should prioritize mechanistic experiments to unveil the intricacies of immune infiltration patterns and elucidate CXCL5's role in drug development. Furthermore, there is a promising avenue for repurposing existing drugs that warrants exploration.

### Supplementary Information

**Additional file 1:** **Supplementary Figure 1.** Results of correlation analysis.

**Additional file 2:** **Supplementary Figure 2.** Figure 2E's original gel image.

## Data Availability

All data in this paper have obtained proof of availability, and original data are available from corresponding authors. The datasets used and/or analysed during the current study available from the corresponding author on reasonable request.
