# Supplementary figures and images for: Prognostic marker CXCL5 in glioblastoma polyformis and its mechanism of immune invasion
Source: BMC Cancer. 2024 Jan 29;24:140. doi: 10.1186/s12885-023-11650-3 (PMC10823677; doi:10.1186/s12885-023-11650-3)

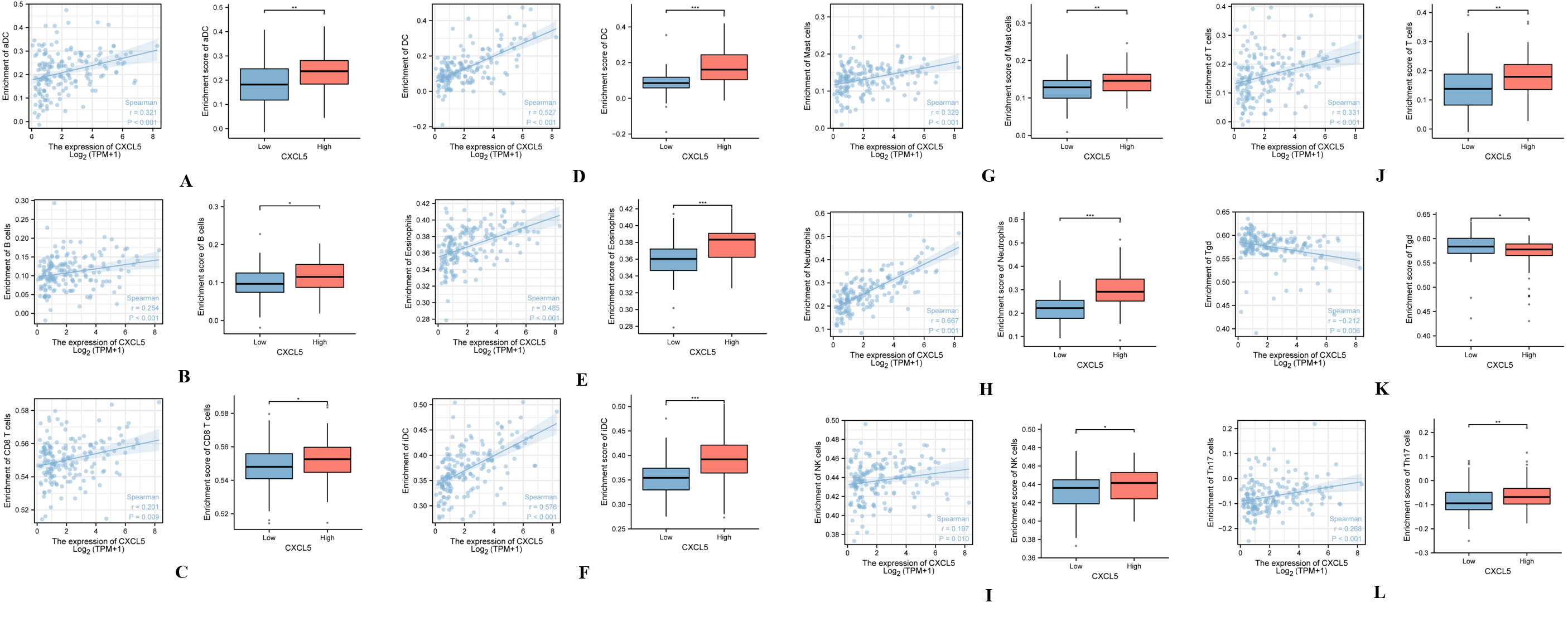

Supplement: Supplementary file 1 — Additional file 1: Supplementary Figure 1. Results of correlation analysis. [file 12885_2023_11650_MOESM1_ESM.tif]

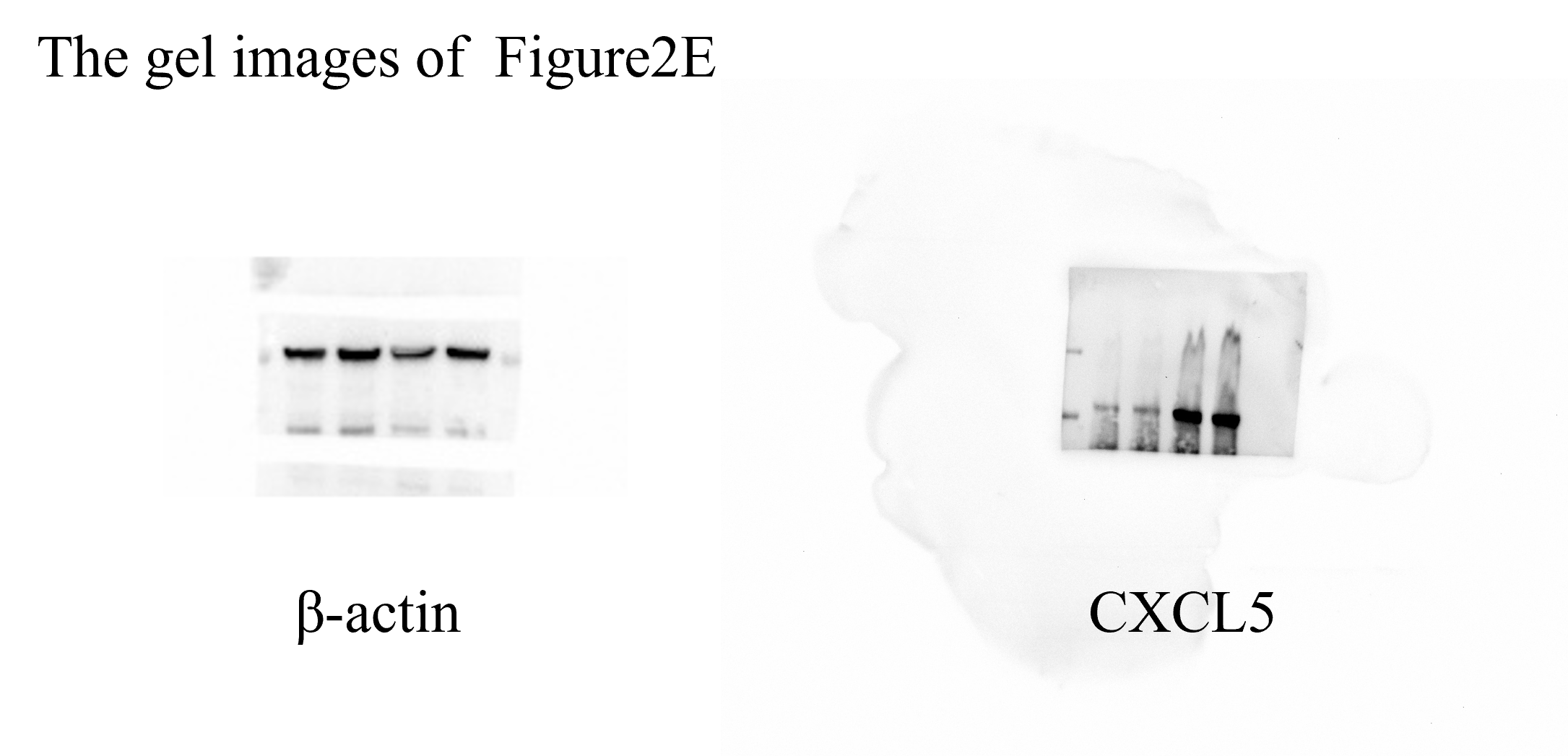

Supplement: Supplementary file 2 — Additional file 2: Supplementary Figure 2. Figure 2E's original gel image. [file 12885_2023_11650_MOESM2_ESM.tif]
